# Development of *Ex Situ* Normothermic Reperfusion as an Innovative Method to Assess Pancreases After Preservation

**DOI:** 10.3389/ti.2022.10038

**Published:** 2022-04-12

**Authors:** Arnau Panisello-Roselló, Emma Folch-Puy, Joan Roselló-Catafau, René Adam

**Affiliations:** ^1^ Experimental Pathology Department, Institute of Biomedical Research of Barcelona (IIBB), CSIC, Barcelona, Spain; ^2^ AP-HP Hôpital Paul Brousse, UR Chronothérapie, Cancers et Transplantation, Université Paris-Saclay, Paris, France

**Keywords:** pancreas, HOPE, normothermia, reperfusion, preservation solutions

Dear Editors,

We read with great interest the paper entitled “*Development of Ex Situ Normothermic Reperfusion as an Innovative Method to Assess Pancreases After Preservation*” (1). After analyzing the *ex situ* assessment of pancreases by normothermic reperfusion (NR), the authors suggested that HMPO2 may be better than SCS; they a further compared two different HMPO2 perfusates: Belzer-MPS and IGL-2.

We would like to point out some considerations concerning this perfusate comparison. Specifically, when the water content in pancreas grafts (as a surrogate for edema assessment) was measured in hypothermic conditions. Under those conditions, IGL2HMP pancreases showed a lower water content than the UWHMP group. These results are concomitant with the lower amylase and lipase levels, well known as injury markers for pancreas in static preservation, which has been validated recently as well in dynamic condition by Branchereau et al. (2). This higher injury prevention exerted by IGL2 would be associated with the water content gain, which in turn is regulated by the presence of oncotic agents such as PEG35 (in IGL2HMP) and HES (in UWHMP). One of the concerns related to pancreas preservation is the development of edema, which is generally regarded as undesirable. Thus, the lower water content in INGL2HMP indicates a better oncotic efficiency of PEG as compared to HES. Moreover, it is widely reported that HES acts as a red blood cell pro-aggregating agent (3), which is a major factor when considering a solution containing PEG to be more suitable for pancreas washout (4).

Recently, we have reported the benefits of that using PEG35 can be beneficial for preventing IRI damage (5) and can have an anti-inflammatory role in acute pancreatitis (6). This is especially relevant for pancreas IR pathophysiology, as a tendency to develop pancreatitis and vascular thrombosis after ischemia has been widely reported (2); notably, these are main causes of early patient morbidity and mortality after pancreas transplantation (7, 8). In addition, IGL2 (PEG35) is very suitable to better preserving luminal glycocalyx deterioration against the mechano-transduction events in hypothermic oxygenated perfusion (HOPE) due to inherent fluid dynamics, whereby the lower viscosity of IGL2 (9) as well as the vasodilatory action derived from NO generation by PEG35 may be relevant factors to be considered (4).

We agree that future investigations are needed to confirm and expand relevant study, especially considering the number of animals used. We highlight the use of a PEG-based solution (IGL2) and its improvement of HOPE strategies, given that the favorable results reported in other solid organs could be extended to pancreas. This would be, for instance, the case of the protection of the mitochondria, as previously reported by us for liver measured as glutamate dehydrogenase (GLDH) (mitochondrial damage) (9, 10).

Especially given the lack of consensus regarding the optimal perfusion solutions and methods for pancreas preservation prior to transplantation, we are grateful for this important paper, as it opens up the dialogue about developing a new paradigm for pancreas preservation.

## Data Availability

The original contributions presented in the study are included in the article/supplementary material, further inquiries can be directed to the corresponding author.
